# A case report of septic shock caused by opportunistic infections associated with anti-interferon-γ autoantibody positivity: diagnostic and therapeutic challenges

**DOI:** 10.3389/fmed.2025.1592152

**Published:** 2025-06-13

**Authors:** Jinlian Shao, Xiao Xu, Xunjie Xie, Yiqi Fan, Dacheng Zhang, Waijiao Tang

**Affiliations:** ^1^Emergency Department, Zhujiang Hospital, Southern Medical University, Guangzhou, China; ^2^Department of Laboratory Medicine, Zhujiang Hospital, Southern Medical University, Guangzhou, China; ^3^Drug Clinical Trial Institution, Department of Pharmacy, Zhujiang Hospital, Southern Medical University, Guangzhou, China; ^4^Department of Respiratory, The Third Affiliated Hospital, Southern Medical University, Guangzhou, China; ^5^Department of Pharmacy, Shenzhen Hospital, Southern Medical University, Shenzhen, China

**Keywords:** anti-interferon-*γ* autoantibody positivity, adult-onset immunodeficiency syndrome, non-typhoidal Salmonella, extraintestinal dissemination, diagnostic and therapeutic thinking

## Abstract

**Background:**

Since 2004, there has been an increasing number of reports on severe, persistent, or recurrent Salmonella infections in adults with adult immunodeficiency associated with anti-gamma interferon antibody positivity (AIGA). AIGA patients experience rapid disease progression upon infection with opportunistic pathogens, high mortality rates, and strong disease latency, posing significant challenges for diagnosis and treatment. This article discusses the diagnosis and treatment strategies for AIGA with opportunistic pathogen infection through the diagnosis and treatment process of a 61-year-old male patient.

**Methods:**

The patient presented with diarrhea and fever for 2 weeks and was diagnosed with non-typhoidal Salmonella infection at an external hospital. The condition progressed to shock and the patient was transferred to our EICU. After admission, the pathogens were confirmed through chest CT, blood culture, blood metagenomic next-generation sequencing (mNGS), and bronchoalveolar lavage fluid (BALF) mNGS, and cell immune function screening and anti-gamma interferon antibody testing were completed. The anti-infective treatment regimen was adjusted based on the test results, and immunoglobulin therapy was administered.

**Results:**

The patient’s blood culture was positive for non-typhoidal Salmonella, and blood mNGS confirmed non-typhoidal Salmonella and *Legionella pneumophila*; BALF mNGS showed *Enterococcus faecium*, *Legionella pneumophila*, *Candida tropicalis*, Candida glabrata, HSV1, and CMV mixed infection. Immune function screening indicated a significant decrease in CD4 + T cells (303 cells/μL) and a significant increase in anti-gamma interferon antibody (163.78 ng/mL), confirming the diagnosis of AIGA. After treatment with meropenem, linezolid, doxycycline, ganciclovir, and caspofungin combined with anti-infective and immunoglobulin therapy, the patient’s condition significantly improved and was discharged.

**Conclusion:**

AIGA patients experience rapid disease progression after infection with opportunistic pathogens. Early identification of anti-gamma interferon antibody and mixed infection pathogens is crucial for treatment.

## Background

Since 2004, there has been an increase in reports of adult-onset immunodeficiency induced by adult-onset immunodeficiency induced by anti-interferon-*γ* autoantibodies (AIGA), which has been identified as a predisposing factor for individuals to develop severe, persistent, or recurrent systemic infections with *Salmonella* species and other pathogens (1). Patients with AIGA who develop opportunistic infections often experience rapid deterioration in their condition and a high mortality rate. The insidious nature of this condition presents a significant challenge to accurate diagnosis and management remain challenging. Identifying characteristic disease patterns and establishing standardized treatment protocols are areas warranting further exploration.

## Case summary

**Figure 1:**
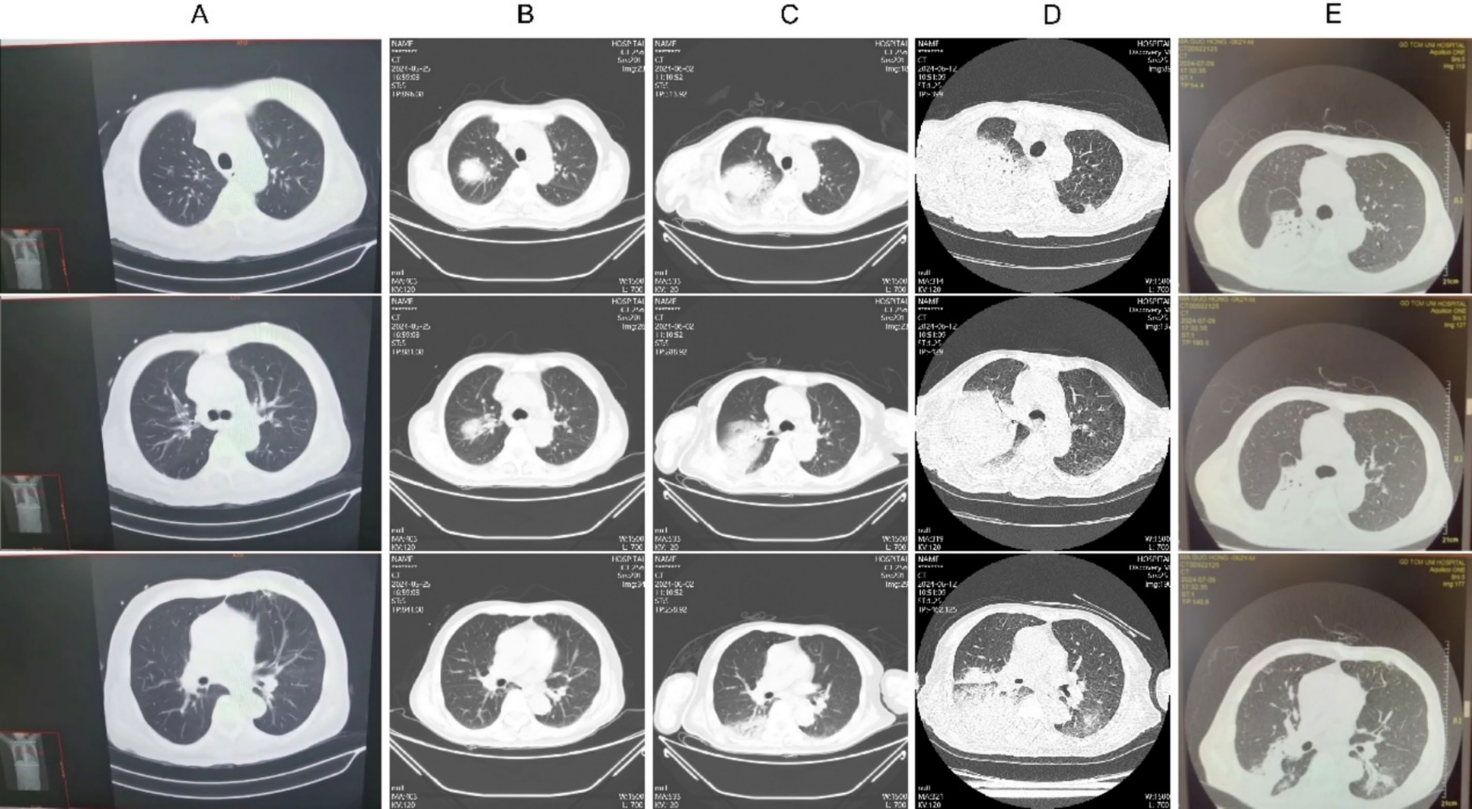
Chest CT imaging alterations during the patient’s clinical management. **(A)** May 14, 2014; **(B)** May 25, w2024; **(C)** June 2, 2024; **(D)** June 12, 2024; **(E)** July 9, 2024.

A 61-year-old male, presented with diarrhea and fever for 2 weeks. Initial testing at an external hospital revealed non-typhoidal Salmonella in blood, urine, and stool samples. A chest CT scan (Figure 1A) showed no abnormalities. Due to the progression to shock, he was transferred to Emergency Intensive Care Unit (EICU) at our hospital. A post-admission chest CT scan (Figure 1B) revealed consolidation in the right lung, and a positive blood culture indicated the presence of non-typhoidal Salmonella. The patient received meropenem, levofloxacin, and azithromycin for infection control, which resulted in a reduction in body temperature, white blood cell count (15.7 to 11.38G/L), procalcitonin (PCT; 179.59 to 47.83 μg/L), C-reactive protein (CRP; 264 to 134 mg/L), and IL-6 (5,000 to 70.4 ng/L). The patient’s condition showed clinical improvement including the resolution of shock and an improvement in stool frequency. However, the platelet count exhibited a persistent decline, dropping from 10 to 3 G/L.

Metagenomic next-generation sequencing (mNGS) of blood samples revealed the presence of *Legionella pneumophila* and *Salmonella*. The patient’s symptoms worsened, with an increase in dyspnea and hypoxemia, and follow-up CT scans (Figures 1C,D) demonstrated a worsening of right lung consolidation and new involvement in the left lower lung. Bronchoalveolar lavage fluid (BALF) was sent for mNGS, which identified the following organisms: *Enterococcus faecium*, *Legionella pneumophila*, *Candida tropicalis*, *Candida albicans*, Herpes Simplex Virus type 1 (HSV-1), and Cytomegalovirus (CMV). Further immunological screening revealed CD4 + T cells at 303/μL, CD8 + T cells at 74/μL, and an HLA-DR positivity rate of 15.38%. Test result of HIV was negative, while anti-IFN-*γ* antibody level was elevated at 163.78 ng/mL, indicating the presence of adult-onset immunodeficiency induced by anti-interferon-γ autoantibodies (AIGA). Treatment with meropenem, linezolid, doxycycline, ganciclovir, and caspofungin, in conjunction with intravenous immunoglobulin (IVIG) at 20 g/day for 5 days, led to gradual absorption of pulmonary infiltrates (Figure 1E), an increase in platelet counts, and significant improvement in overall condition, thereby allowing discharge.

## Discussion

Extraintestinal dissemination of non-typhoidal Salmonella is commonly observed in immunocompromised individuals. Studies have shown that in Southeast Asia, 88% of individuals with multiple opportunistic infections have detectable anti-IFN-*γ* autoantibodies (2). Consequently, an anti-IFN-γ antibody test was conducted, which returned positive. The absence of a distinctive phenotype makes early diagnosis of AIGA a challenge. In this patient, HIV was negative, and there was no history of long-term immunosuppressive therapy, corticosteroid use, malignancy, or autoimmune disorders. The presence of extraintestinal dissemination of Salmonella and multiple opportunistic infections indicated the possibility of AIGA. This prompts the question of whether non-typhoidal Salmonella-infected patients with extraintestinal spread should be screened for anti-IFN-*γ* antibody levels for early diagnosis and targeted treatment.

The patient’s initial lung consolidations were presumed to be due to Salmonella or Legionella pneumonia. However, as lesions progressed despite treatment, mNGS confirmed the involvement of multiple opportunistic pathogens. This highlights that, even if blood cultures are positive for Salmonella, suboptimal response to treatment in such patients warrants early use of diverse diagnostic methods to confirm the presence of alternative opportunistic infections. Additionally, treatment for the primary disease is critical. Based on related literature (3), immunosuppressants or corticosteroids were deemed unsuitable for this patient after evaluation. Thus, a five-day IVIG regimen of 20 g/day was administered. The patient experienced no recurrence of infection, and pulmonary imaging gradually improved, ultimately leading to recovery and discharge. However, since the patient has AIGA and belongs to a high-risk group for opportunistic pathogen infections, there is a significant likelihood of recurrent infections caused by opportunistic pathogens such as NTM (Nontuberculous Mycobacteria), Salmonella, and Legionella in the future. The patient should closely monitor their immune status and seek medical attention promptly if any abnormalities are detected. It is imperative to acknowledge that our study is subject to certain methodological constraints that warrant explicit disclosure. For example, we need to dynamically monitor the serum IFNγ antibody concentration and IFNγ level of AIGAs patients, and preferably measure the level of anti-IFNγ neutralizing antibodies.

## Conclusion

Patients with AIGA often experience rapid disease progression upon acquiring opportunistic infections. Timely differentiation of AIGA and early identification of additional opportunistic pathogens beyond Salmonella, coupled with appropriate immunotherapy, are crucial for the effective treatment of this patient population.

## Data Availability

The original contributions presented in the study are included in the article/supplementary material, further inquiries can be directed to the corresponding authors.

## References

[1] Zhang B Fan J Huang C Fan H Chen J Huang X. Characteristics and outcomes of anti-interferon gamma antibody-associated adult onset immunodeficiency. J Clin Immunol. (2023) 43:1660–70. doi: 10.1007/s10875-023-01537-0, PMID: 37365453 PMC10499688

[2] Browne SK Burbelo PD Chetchotisakd P Suputtamongkol Y Kiertiburanakul S Shaw PA. Adult-onset immunodeficiency in Thailand and Taiwan. N Engl J Med. (2012) 367:725–34. doi: 10.1056/NEJMoa1111160, PMID: 22913682 PMC4190026

[3] Qiu Y Fang G Ye F Zeng W Tang M Wei X. Pathogen spectrum and immunotherapy in patients with anti-IFN-gamma autoantibodies: a multicenter retrospective study and systematic review. Front Immunol. (2022) 13:1051673. doi: 10.3389/fimmu.2022.1051673, PMID: 36569827 PMC9772057

